# Notes on Sciatica

**Published:** 1892-03

**Authors:** B. Simmons

**Affiliations:** Sidney, New South Wales


### NOTES ON SCIATICA.

B. Simmons, M. D., Sidney, New South Wales.

The following notes on Sciatica together with the repertory were originally compiled for my own use. They have proved of immense service to me ; so I indulge the hope that they may be helpful to others.

Some of the most brilliant successes that our school has achieved have been in the treatment of the above-named malady, for the cure of which allopathy appears powerless. Indeed it is only by the administration of the most carefully selected homœopathic remedy that satisfactory results may be obtained, My own experience is strongly in favor of the higher and highest potencies administered in the way recommended by our most careful observers, and most faithful followers of Hahnemann. The following *accidental* cure of a chronic case of sciatica by the administration, subcutaneously, of three doses of Osmic acid (100th of a grain being given on each occasion) is worth recording.

A. B., aged forty years, male, disease began on the left side, then went over to the right, when situated in the former position body was drawn over somewhat to the left side, pains aggravated by sitting, relieved by standing and walking, when the *right side* became involved there was a settled burning up the left leg to left hip, there were also shooting pains *upward* alternating with the burning, sensation of band of iron round calf of leg, stiffness of affected part, loss of power, and general emaciation, at times sensation of pricking in the toes, *general aggravation* from cold at night in bed, from lying on painful right side, from any jar of the bed, in wet weather. The case had lasted about two years, the cure was effected in a comparatively short space of time.

### NOTES ON SCIATICA.

B. Simmons, M. D., Sydney, New South Wales.

*Aconite.*—Aggravation from chill in cold air, with restlessness without relief.

*Ammon-mur.*—Sensation of tension. Aggravation when sitting.

*Arsenicum.*—Shooting pain, burning pain like fire, with great restlessness without relief, heat relieves; better by flexing limb.

*Belladonna.*—Pain as if beaten. As if the bone was carious. Gnawing in the bones. Tearing pains going upward. Aggravation when sitting, better by warmth, standing, becoming erect, walking. Restlessness of legs.

*Berberis.*—Aggravation from changes of weather, and before boisterous winds.

*Byronia.*—Aggravation from *motion*, *least motion*, from sitting up and in the evening, better by cold applications and rest and by lying on painful side.

*Capsicum.*—Shooting, tearing pains. Aggravation when coughing.

*Chamomilla.*—Drawing, tearing, pains, cramp-like tension of the muscles, left side chiefly, with marked irritability.

*Coffea.*—Rending, shooting pains. Aggravated when walking in the afternoon, at night.

*Colocynth.*—Crampy pain, as if screwed in a vice, pains shooting downward like lightning, psoas muscle feels too short, drawing pain extending to knee, muscles of the thigh stiff and tight as if screwed up, sensation as of a band of iron round part. Aggravation in the evening and when walking. Better by stooping forward, lying on painful side, and by drawing up the leg; right limb chiefly affected.

*Drosera.*—Pressing pain, aggravated from pressure, from stooping, and from lying on the painful part, better after rising from bed.

*Ferrum.*—Aggravation in the evening until twelve P. M., better by walking, restless, must get up and walk.

*Gnaphalium.*—Intense pain along the sciatic nerve, numbness alternates with pain. Aggravation walking.

*Ignatia.*—Lancinating, cutting pains, beating, bursting pains, aggravated in winter, better in summer, chilliness with thirst, flushes of heat, chiefly face, without thirst.

*Iris.*—Shooting pain, pain as if wrenched, sensation as of dislocation. Aggravation sitting down and gentle motion. Better by violent motion. Left hip affected.

*Kali-bich.*—Pains extend to the knee, left side affected. Aggravated from pressure.

*Kali-carb.*—Crampy, tearing pains, pains as if bruised. Aggravation from movement. During sleep pains felt.

*Kali-iod.*—Gnawing in the bones, darting in left hip, tearing in the right thigh and knee. Aggravation when walking, at night, lying on painful side or back.

*Kreosote.*—Pain as if dislocated, in left thigh, boring pains alternate with numbness, leg feels too long when standing.

*Lac-can.*—Pains change frequently *from one side to the other.*

*Ledum.*—Pressure in the right hip, pains going upward. Aggravation from motion, heat of the bed, in the evening till twelve P. M.

*Lachesis.*—Pain as from a hot iron, right side, left side. Aggravation when sitting, walking, after sleeping, rising up erect. Better by lying still in bed.

*Lycopodium.*—Tension in the left hip, with jerking of the limbs. Aggravation every four days.

*Natrum-sulph.*—Piercing pain in the left hip, right side also affected. Aggravation, stooping, rising from a seat, moving in bed, lying long in one position, rest. Better by motion (left hip).

*Nux-vom.*—Pains darting upward, numbness. Aggravation in the morning, turning the body round; night, lifting; motion, during stool. Better from applications of hot water.

*Phytolacca.*—Shooting pains, cutting pains, drawing pains; right side, pains go downward. Aggravation at night.

*Plumbum.*—Drawing, pressing pains, with muscular atrophy, with exhaustion from walking. Aggravation, walking.

*Podophyllum.*—Tenderness to pressure, left hip. Aggravation, ascending.

*Psorinum.*—Dislocation pain, tension, arms weak. Aggravation, walking.

*Rhus.*—Numbness. Aggravation, over-exertion, night, rising from bed, rising from a seat, cold or damp weather. Better by rubbing, heat, becoming warm from exercise, from continued motion, change of position. Right side affected, restlessness, with relief for a short time by movement.

*Rhododendron.*—Aggravation in stormy weather.

*Ruta.*—Bones feel bruised, hamstring muscles feel shortened, broken feeling in the bone. Aggravation, beginning to move, rising up, sitting, lying, better by walking. Restlessness, must continue walking, sleep prevented thereby.

*Sepia.*—Lancinating stitches. Aggravation, rising up, three to five A. M. Better after rising from bed, by walking slowly, and during pregnancy. Veins swollen.

*Stramonium.*—Left side, drawing pain in thigh and knee.

*Valerian.*—Pain as if the thigh would break. Aggravation, standing.

*Tellurium.*—Right side. Aggravation, during stool, coughing, laughing, lying on the painful side.

### REPERTORY.

*Band, around part, like an iron.* Coloc.
*Beaten or bruised, as if.* Bell., Kali.
— — *as if bone were.* Ruta.
*Bone, pains in.* Bell., Kali-hyd., Ruta, Valer.
— — *as if broken.* Ruta.
— *break, as if the thigh would.* Valer.
— *as if carious.* Bell.
— *gnawing, shooting.* Bell.
— — *in.* Kali-hyd.
— *bruised sensation.* Ruta.
*Boring.* Kreos.
*Break, as if thigh would.* Valer.

*Broken, feeling in bone.* Ruta.
*Bruised, sensation in bone.* Ruta.
*Burning like a hot iron.* Lach.
*Bursting, beating.* Ignat.
*Carious, as if the bone were.* Bell.
*Contraction or constriction, as from an iron band.* Coloc.
— — *as if screwed up tightly.* Coloc.
— — — *the psoas was too short.* Coloc.
— — — *hamstrings were too short.* Ruta.
— — *as from tension, or as if too tight.* Acon., Lyc., Psoric.
*Cramp-like tension.* Cham.
*Crampy tearing.* Kali.
*Crampy, as if in a vice.* Coloc.
*Cutting.* Phyto.
— *lancinating.* Ignat.
*Darting in left hip.* Kali-iod.
*Dislocation, pain as from.* Iris., Kreos.
— — *in left hip.* Psorin.
*Drawing pain.* Phyto.
— *pressing.* Phyto.
— *tearing.* Cham.
— *to knee.* Coloc.
— *to left knee.* Stram.
— *wrenching.* Iris.
*Drawing.* (See also *Tearing.*)
*Gnawing in bone.* Kali-iod., Ruta.
— *shooting in bone.* Bell.
*Hamstrings feel too short.* Ruta.
*Intense pain along sciatic nerve.* Gnaphal.
*Lancinating, cutting.* Ignat.
*Long, leg feels too.* Kreos.
*Numbness.* Nux, Rhus.
— *alternates with pain.* Graph., Kreos.
*Piercing in left hip.* Nat-sulph.
*Pressing pain.* Dros.
— — *in right hip.* Ledum.
— — *drawing.* Phyto.

*Psoas muscle feels too short.* Coloc.
*Rending, shooting.* Coffea.
*Shooting.* Arsen., Bry., Iris, Phyto.
— *gnawing in bone.* Bell.
— *lancinating.* Sepia.
— *like lightning downward.* Coloc.
— *tearing.* Caps., Coffea.
— *rending.* Coffea.
— (*darting in left hip*). Kali-iod.
— (*lancinating cutting*). Ignat.
— *piercing in left hip.* Nat-sulph.
*Short, psoas feels too.* Coloc.
— *hamstrings feels too.* Ruta.
*Screwed up tightly, as if.* Coloc.
*Tearing.* Bell.
— *shooting.* Caps., Coffea.
— *drawing.* Cham.
— *crampy.* Kali.
— *in right thigh.* Kali-iod.
— — *left thigh.* Stram.
— (*wrenching*). Iris.
— (See also *Drawing.*)
*Tenderness to pressure.* Pod.
*Tension, feeling of.* (See also *Contraction.*)
*Tension.* Acon., Lyc., Psor.
*Vice, as if in a.* Coloc.
— *crampy, as if in a.* Coloc.
*Wrenching.* (See also *Drawing and Tearing.*) Iris.

DIRECTION OF PAINS.

*Downward.* Phyto.
— *drawing to knee.* Coloc.
— *shooting.* Coloc.
— *to knee.* Kali-bich.
*Upward.* Bell., Ledum, Nux.
— *darting.* Nux.
*Changing from side to side.* Lac-can.

CONDITIONS OF AGGRAVATION.

*Ascending.* Podo.
*Bed, moving in.* Natrum-sulph.
— *turning in.* Natrum-sulph.
— *rising from.* Rhus.
*Cold air, from chill in.* Acon.
*Coughing.* Capsicum, Tellurium.
*Erect, becoming.* Bry., Lachesis, Ruta, Sepia. (See *Rising from Bed, Rising from Sitting.*)
*Exertion, from over.* Rhus.
*Heat, of bed.* Ledum.
*Laughing.* Tellurium.
*Lifting.* Nux.
*Lying on painful side.* Drosera, Kali-iod., Tellurium.
— *long in one position.* Natrum-sulph.
— *on back.* Kali-iod.
— *whilst.* Ruta.
*Motion.* Bry., Kali, Ledum, Nux. (See *Walking, etc.*)
*Move, beginning to.* Ruta.
*Pressure.* Drosera, Kali-bich., Podophyllum.
*Rest, during.* Natrum-sulph., Ruta, Rhus. (See *Lying, etc.*)
*Sitting.* Ammon-mur., Bell., Lach., Ruta.
— *up, on.* Bryonia.
— *down, on.* Iris.
— *rising from.* Natrum-sulph., Rhus.
*Sleep, after.* Lachesis, Kali-iod.
— *during, pains felt.* Kali.
*Sneezing.* Tellurium.
*Standing.* Kreosote, Valer.
*Stool, during.* Nux, Tellurium.
*Stooping.* Drosera, Natrum-sulph.
*Turning round when walking.* Nux. (See also *Turning in Bed.*)
*Walking.* Coffea, Colocynth, Graph., Lach., Kali-iod., Podo.
— *slowly.* Iris.

*Weather, change of.* Berberis.
— *during stormy.* Rhodod.
— *cold, dry.* Acon.
— *cold, damp.* Rhus.
— *before windy.* Berb.
*Winter, in.* Ignat.

### TIMES OF AGGRAVATION.

*Morning.* Nux.
— *three to five* A. M. Sepia.
*Afternoon.* Coffea.
*Evening.* Bry., Coloc, till midnight, Ferrum, Ledum.
*Night.* Coffea, Kali-iod., Nux, Phyto., Rhus.
*Every four days.* Lycop.

### CONDITIONS OF AMELIORATION.

*Bed, after arising from.* Drosera, Sepia.
*Cold.* Bry.
— *applications.* Bry.
*Drawing up limb.* Ars., Coloc.
*Erect, becoming.* Bell.
*Exertion, violent.* Iris.
*Flexing limb.* Ars., Coloc.
*Heat.* Bell., Nux, Rhus.
— (*warm applications*). Nux, Rhus.
— *in summer.* Ignat.
— (*warm from exercise*). Rhus.
*Lying on painful side.* Bry., Coloc.
— *still in bed.* Lach. (See *Rest.*)
*Motion, amel.* Nat-sulph., Rhus, Ruta.
— *violent, amel.* Iris.
— *continued amel.* Rhus, Ruta. (See *Walking.*)
*Pregnancy, during.* Sepia.
*Pressure.* Coffea.
*Rubbing.* Rhus.
*Rest, during.* Bry., Lach.

*Standing.* Bell.
*Summer.* Ignat.
*Stooping.* Coloc.
*Walking.* Bell., Ferrum, Rhus, Ruta, Sepia.
— *slowly.* Sepia.
— *quickly.* Iris.
— *continued.* Rhus, Ruta.
*Weather (summer).* Ignat.

CONCOMITANTS.

*Diarrhœa, alternating with.* Psorin.
*Fatigue, great from walking.* Plumb.
*Limbs, jerking of.* Lyc.
*Muscular atrophy.* Plumb.
*Restlessness, with relief from movement.* Rhus.
— *without relief from movement.* Ars., Ferrum, Ruta.
— *of legs.* Bell.
*Veins, swollen.* Sepia.

NOTE.—In prescribing for a case of sciatica, the concomitants —that is, all the other subjective symptoms of the patient, must be included in the picture.

